# Comprehensive management of chronic insomnia in Spain: a multidisciplinary modified RAND/UCLA consensus with recommendations on daridorexant

**DOI:** 10.3389/fpsyt.2026.1927424

**Published:** 2026-10-13

**Authors:** Ainhoa Álvarez-Ruiz-Larrinaga, Lorenzo Armenteros, Carmen Bellido, Laura Lillo Triguero, José Martínez-Raga, Julio Pascual, Luis San

**Affiliations:** 1 Neuroscience, Sleep Disorders, Bioaraba Health Research Institute, Vitoria-Gasteiz, Spain; 2 Osakidetza Basque Health Service, Araba University Hospital, Vitoria-Gasteiz, Spain; 3 Primary Care, Centro de Salud Islas Canarias, Lugo, Spain; 4 Spanish Society of General and Family Physicians (SEMG), Madrid, Spain; 5 Occupational Risk Prevention Department. General University Hospital of Castellón, Castellón, Spain; 6 Spanish Association of Occupational Medicine, Madrid, Spain; 7 Codirector of Sleep Unit, Neurology Service, Ruber International Hospital, Madrid, Spain; 8 Neurology Service, Getafe University Hospital, Madrid, Spain; 9 Service of Psychiatry And Mental Health, University Hospital Doctor Peset, Valencia, Spain; 10 Service of Neurology, University Hospital Marqués de Valdecilla, Universidad de Cantabria and IDIVAL, Santander, Spain; 11 Senior Psychiatrist, Miembro del CIBERSAM, Colegio de Psiquiatras Eméritos de la Sociedad Española de Psiquiatría y Salud Mental, Madrid, Spain

**Keywords:** chronic insomnia, daridorexant, daytime functioning, hyperarousal, dual orexin receptor antagonists, sleep architecture

## Abstract

**Introduction:**

Chronic insomnia is a prevalent 24-hour disorder with significant daytime impact. Its management is frequently complicated by conceptual barriers, limited use of standardised diagnostic tools and prolonged benzodiazepine use, while insomnia is often overlooked or inadequately coded as an independent disorder. These challenges highlight the need for improved recognition and clearer therapeutic pathways. Dual orexin receptor antagonists (DORAs), particularly daridorexant, offer a new pharmacological option targeting wake-promoting pathways, yet practical guidance on their clinical use remains limited.

**Method:**

A multidisciplinary panel of seven experts conducted a national consensus using the RAND/UCLA Appropriateness Method. Seventy-four statements addressing the nature, diagnosis and treatment of chronic insomnia, including the clinical use of daridorexant, were evaluated across two rating rounds and a structured discussion. Consensus was reached on 36 statements in round one; one statement was removed; and the remaining 37 achieved consensus in round two.

**Results:**

Experts agreed that chronic insomnia is a distinct disorder, with hyperarousal as a central mechanism, and emphasised the importance of targeting daytime functioning and preserving sleep architecture. Daridorexant was considered appropriate across heterogeneous clinical profiles, supported by evidence demonstrating improvements in nocturnal sleep, daytime functioning and sleep architecture, along with favourable tolerability and low abuse potential.

**Conclusions:**

This consensus supports a pragmatic and patient-centred approach to chronic insomnia management, integrating current evidence with expert clinical experience. The recommendations may help guide decision-making in routine practice, including the use of daridorexant when pharmacological treatment is considered appropriate.

## 1 Introduction

Chronic insomnia is a highly prevalent and impairing sleep disorder, affecting approximately 10% of adults in Europe (1), and around 6.4% of the population in Spain (2), although recent epidemiological studies suggest that the prevalence of chronic insomnia may be considerably higher (14%) (3). It is characterised by persistent difficulties in initiating or maintaining sleep, or experiencing non-restorative sleep, despite having adequate opportunities and circumstances to sleep, for at least three nights per week for a minimum of three months (4–6). This leads to daytime dysfunction and subsequent impairment of quality of life (7, 8).

International guidelines from the European Sleep Research Society (1) and the American Academy of Sleep Medicine (9) underscore the need for a comprehensive and multimodal treatment strategy. Cognitive Behavioural Therapy for Insomnia (CBT-I) remains the recommended first-line intervention. However, despite its well-established efficacy, its implementation in real-world clinical practice is limited. Barriers include system-level constraints (e.g. scarcity of specialists and resources), lack of trained clinicians and awareness, and patient-related factors (e.g. low demand or poor adherence to intensive therapy) (10–12). Furthermore, even when adequately delivered, some patients may require pharmacotherapy if their response to CBT-I is partial or insufficient (1, 9). Consequently, pharmacological treatment remains a common component of clinical management.

Available pharmacological treatments include positive GABAergic modulators specifically benzodiazepines (BZDs) and benzodiazepine receptor agonists (“Z-drugs”), antidepressants, antipsychotics, antihistamines, melatonin receptor agonists, and dual orexin receptor antagonists (DORAs) (13). Clinical recommendations suggest short-term use (≤4 weeks) of classical BZDs, Z-drugs, and sedative antidepressants. Antihistamines, antipsychotics, and herbal preparations are not recommended due to limited efficacy and adverse effects, although these drugs are widely used off-label in clinical practice (14). Widespread prescription of melatonin is also not advised except in cases of circadian rhythm disorders (15).

Among pharmacological options, DORAs represent the most recent class of insomnia medication. They are considered to represent a new generation of drugs, with a mechanism of action that is distinct from that of traditional sedative–hypnotic drugs. Rather than leading to sedation, these agents suppress wakefulness by inhibiting orexin-mediated signalling pathways. Specifically, they prevent orexin-A and orexin-B neuropeptides from binding to their respective orexin receptors, which results in reduced arousal and maintenance of sleep. Because orexin neurons are confined to the hypothalamus, DORAs act on a more circumscribed neuroanatomical target than other classes of hypnotic medications (16, 17). Daridorexant, currently the only DORA approved in the European Union (EU) for chronic insomnia, has emerged as an important therapeutic option (1). Several randomised and observational studies have demonstrated its efficacy and effectiveness in significantly improving sleep and daytime functioning in the short and long term (18–26). Due to its safer profile and absence of misuse risks, daridorexant is the only pharmacological option receiving a grade A recommendation for treatment beyond 4 weeks in the European Insomnia Guidelines when CBT-I is unavailable or ineffective (1).

In Spain, additional challenges further complicate insomnia management. Conceptual barriers and the lack of standardised diagnostic tools contribute to prolonged BZD use, despite guidelines advising restricted duration due to risks of dependence and adverse outcomes (27, 28). Moreover, insomnia is frequently overlooked and inadequately coded as a primary disorder. This is evident from the discrepancy between its high prevalence in the general population and the low proportion of diagnoses recorded in primary care (29). Indeed, Spain continues to rank among the highest worldwide in BZD prescription rates (30, 31). This pattern reflects a broader international concern, as BZD are increasingly regarded as “special risk” medications due to their cognitive, psychomotor and withdrawal-related liabilities, particularly with long-term use (32). These factors highlight the need for improved recognition of insomnia as an independent clinical condition and for therapeutic strategies.

Moreover, as daridorexant was recently approved in the EU, uncertainties remain regarding its implementation in routine clinical practice. In light of this, an Italian expert group has recently published a consensus document addressing the use of daridorexant in clinical practice (33), and more recently, European experts in sleep and neuropsychopharmacology have developed guidelines for switching or deprescribing hypnotic medications in chronic insomnia (34).

Given the growing interest in wake-promoting pathway modulation and the need for clearer guidance on the use of daridorexant in clinical practice, this consensus project aimed to evaluate expert agreement on key aspects of the nature, diagnosis, and management of chronic insomnia, with special focus on the appropriate use of daridorexant to support clinicians within the Spanish healthcare system in their day-to-day management of patients with chronic insomnia.

## 2 Methods

### 2.1 Study design

This national, non-interventional consensus study was conducted using a modified RAND/University of California, Los Angeles (UCLA) Appropriateness Method (35), a structured process combining the best available evidence with expert clinical judgment to assess the appropriateness of statements in areas where evidence is limited or controversial. The process employs sequential rounds of rating and discussion to generate quantitative and qualitative consensus among experts (**Figure 1**).

**Figure 1:**
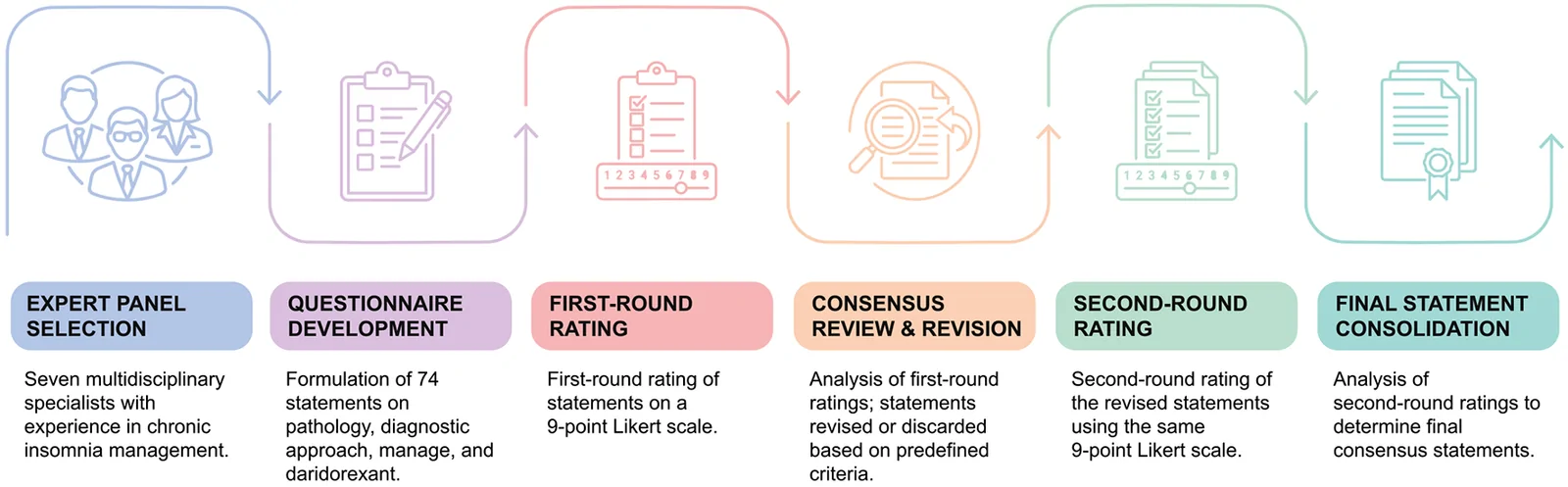
Scheme of the study design based on the RAND-UCLA methodology.

The objective of this consensus project was to evaluate the appropriateness of a series of statements related to the nature, diagnosis, and treatment of chronic insomnia, as well as aspects specific to the use of daridorexant. The study involved two rounds of evaluation followed by a final discussion and synthesis of recommendations. The primary objective of the exercise was to determine whether predefined consensus thresholds had been reached. Therefore, agreement percentages and mean ratings were selected as summary measures of endorsement.

To ensure impartiality, the study was conducted with the support of an independent entity, which provided meeting and methodological facilitation, material preparation and scientific accuracy. The sponsor (Idorsia Pharmaceuticals) had no role in the rating process, consensus voting, interpretation of individual ratings, or final approval of the manuscript. Ratings were completed independently and anonymously by panel members through online questionnaires. All authors disclosed relevant financial and professional relationships, which are reported in the Conflict-of-Interest section.

As the study did not include human participants or patient data and consisted solely of expert opinion collection, institutional review board approval was not required. All participants voluntarily agreed to participate in the consensus process.

### 2.2 Participants and statement development

The expert panel was selected by invitation, targeting clinicians with recognised expertise in sleep medicine, substantial experience in managing insomnia, and regular prescribing of hypnotic drugs and daridorexant in routine practice. The selection also aimed to ensure multidisciplinary representation and national coverage of the main clinical settings involved in insomnia management in Spain. The panel comprised physicians from several specialties key to the management of insomnia, including neurology (n=2), psychiatry (n=2), clinical neurophysiology (n=1), occupational medicine (n=1), and primary care (n=1). In the initial phase, the experts identified three major thematic domains to guide the scope and structure of the consensus (**Table 1**). Based on these predefined domains, a targeted non-systematic literature review was subsequently conducted to identify evidence relevant to the development of the consensus statements. Relevant evidence sources included the most recent European Insomnia Guideline and expert consensus documents on the diagnosis and treatment of insomnia, as well as narrative reviews addressing pathophysiology and therapeutic strategies. In addition, clinical evidence was considered from clinical trials, pooled analyses, and meta-analyses evaluating the efficacy and safety of daridorexant. Observational and real-world studies were also included to complement trial data and provide insights into long-term effectiveness and tolerability in routine clinical practice.

**Table 1:** Domains used to draft the consensus statements.

Domain	Description
Domain 1	Nature and pathophysiology of chronic insomnia (e.g., symptoms versus disease entity, role of hyperarousal, therapeutic targets, comorbidity relationships).
Domain 2	Management and treatment considerations (e.g., sleep induction versus wake inhibition, daytime functioning, sleep architecture, and dependence potential of DORAs).
Domain 3	Clinical use of daridorexant (e.g., comparative efficacy, optimal duration, adherence, responder profiles, and treatment switching).

DORAs, dual orexin receptor antagonists.

Next, 74 statements were formulated which were organised according to the three domains. The participating experts reviewed and helped draft the statements questionnaire.

### 2.3 Consensus rating procedure

The consensus process consisted of two anonymized online rating rounds conducted using Microsoft Forms. In both rounds, experts rated their level of agreement with each statement using a 9-point Likert scale, where 1 indicated “completely disagree” and 9 “completely agree”. In the first round, each expert independently rated all items based on clinical experience and current evidence. Ratings for each item were categorized as rejected (scores 1–3), uncertain (4–6), or accepted (7–9). For each item, the proportion of experts assigning scores in each category was calculated. Items with ≥75% of ratings in the 1–3 range were classified as rejected (unless a specific reason justified re-evaluation), those with ≥75% of ratings in the 7–9 range were classified as accepted, and those with ≥75% of ratings in the 4–6 range were considered uncertain. In a seven-member panel, this corresponded to agreement by at least 6 experts (85.7%), thereby ensuring that consensus was only achieved when a qualified majority of experts endorsed a given statement. After the online rating phase, a virtual meeting was held to present and discuss the distribution of scores. The discussion meetings were moderated by the methodological coordinating team, which facilitated discussion and statement refinement but did not participate in the rating process. During this meeting, items that had not reached the predefined consensus thresholds, as well as statements judged to be ambiguous, overlapping or redundant, were reviewed in detail and, when appropriate, modified, clarified or merged before being re-submitted to the panel in the subsequent rating round. Modifications were limited to clarifying wording, reducing ambiguity, eliminating redundancy, or incorporating comments raised during panel discussion. No new concepts were introduced after completion of Round 1.

In the second round, the revised statements were rated using the same 9-point Likert scale and classification thresholds. Items reaching the predefined consensus criteria in the second round were classified as accepted or rejected and were incorporated accordingly into the final consensus statement set. The final results were then shared and discussed in a subsequent online meeting held in September 2025, during which the panel reviewed the agreed statements and supported the drafting of the present manuscript.

## 3 Results

All seven experts took part in the consensus process, voting on the statements and participating in the subsequent discussion meeting.

In round 1, experts reviewed 74 statements (17 related to the first domain, 20 to the second domain, and 37 to the third domain), reaching consensus on 36 statements (48.6%), all with 100% agreement in the acceptance range.

During the panel meeting, 1 statement was removed after discussion. The statement: “Patients with insomnia related to hyperarousal may derive greater benefit from treatment with daridorexant for maintenance insomnia” was excluded because the panel reached consensus that they did not agree with it. In addition, the statement was considered inconsistent with two related items addressing hyperarousal and the definition of a responder patient profile. In round 2, the remaining 37 items from the initial 74 were evaluated after modifications agreed upon in the round 1 meeting and re-submitted to the expert group for reassessment. This phase confirmed and consolidated previous agreements, achieving unanimous consensus (100%) on 35 out of 37 statements and strong consensus (≥85%) on two statements. The mean appropriateness score of the items included in the final document ranged from 8.29 to 9.00, reflecting a very high level of agreement across all analysed domains. No items remained in the rejected or uncertain categories at the end of the process. The statements, ordered by domain are reported in **Tables 2**–**4** and presented in the following paragraphs. The key consensus conclusions are presented in **Figure 2**.

**Table 2:** Consensus statements on the pathophysiology and diagnostic approach to chronic insomnia.

#	Statement	Phase (consensus reached)	%	Mean	SD	Median
A. Nature of Chronic Insomnia: Symptoms versus Distinct Clinical Entity						
1	Chronic insomnia should be considered a clinical disorder per se when it meets the diagnostic criteria defined in clinical guidelines (ICSD-3, DSM-5, ICD-11), regardless of the presence of comorbidities.	1	100%	8.86	0.38	9
2	The presence of daytime manifestations (fatigue, excessive sleepiness, cognitive and functional impairments, and/or mood disturbances), which is a prerequisite for the diagnosis of insomnia, constitutes one of the main reasons for consultation in this disorder.	1	100%	8.43	0.98	9
3	Given its transdiagnostic nature, insomnia should be regarded as a distinct clinical entity that may coexist with other sleep–wake disorders, medical or psychiatric conditions, or substance use (medications, alcohol, caffeine, nicotine, or other drugs), and requires a specific management approach.	1	100%	8.71	0.49	9
4	Recognition of chronic insomnia as a differentiated clinical entity enables the implementation of targeted treatment strategies.	1	100%	9.00	0.00	9
B. Hyperarousal as an Underlying Mechanism						
5	Cognitive and emotional hyperarousal is the predominant underlying cause of chronic insomnia.	1	100%	7.86	0.69	8
6	Hyperarousal should be assessed as part of the diagnostic evaluation of chronic insomnia.	2	85.7%	8.29	1.11	9
7	The presence of hyperarousal may explain the limited response to traditional hypnotics in some patients.	2	100%	8.86	0.38	9
C. Sleep Quality versus Quantity as a Therapeutic Goal						
The therapeutic approach to chronic insomnia should consider:						
8	– Improvement in sleep quantity (e.g., total sleep time).	1	100%	8.57	0.79	9
9	– Improvement in patient-perceived sleep quality (how restorative and refreshing the sleep experience is).	1	100%	9.00	0.00	9
10	– Improvement in daytime functioning.	1	100%	8.86	0.38	9
The following therapeutic outcomes should be prioritised over simply increasing total sleep time:						
11	– Enhancement of subjective sleep quality (how restorative and refreshing the sleep experience is).	1	100%	8.57	0.53	9
12	– Improvement in daytime functioning.	1	100%	8.57	0.53	9
D. Bidirectional Relationship between Chronic Insomnia and Comorbidities						
13	Chronic insomnia may act as a risk factor for the development or worsening of medical and psychiatric comorbidities, underscoring the importance of a specific treatment approach.	1	100%	9.00	0.00	9
14	Medical or psychiatric comorbidities can trigger sleep disorders, particularly chronic insomnia.	2	100%	8.71	0.49	9
15	Insomnia symptoms may persist or worsen despite treatment of the associated comorbidities.	1	100%	8.86	0.38	9
16	Pharmacological treatments prescribed for comorbidities may induce insomnia symptoms.	1	100%	8.71	0.49	9
17	The relationship between chronic insomnia and comorbidities is bidirectional and may change over time, making appropriate follow-up and separate, specific management of both conditions necessary.	1	100%	8.86	0.38	9

SD, standard deviation.

**Table 3:** Consensus statements on the management and treatment of chronic insomnia.

#	Statement	Phase (consensus reached)	%	Mean	SD	Median
A. Sleep Induction versus Wakefulness Inhibition						
1	Selective inhibition of the orexinergic pathway, through the blockade of OX1R and OX2R receptors, reduces neuronal hyperactivity responsible for wakefulness and facilitates sleep onset while preserving its physiological architecture, in contrast to nonspecific sedative mechanisms.	1	100%	8.71	0.49	9
2	Specific suppression of wake-promoting signals through dual orexin antagonists represents a strategy more aligned with sleep physiology than direct sleep induction via GABAergic activation or inhibition of other neurotransmitter systems (histaminergic, cholinergic, noradrenergic, serotonergic, or dopaminergic).	2	100%	8.57	0.79	9
3	The treatment of chronic insomnia should focus on modulating systems that maintain wakefulness, promoting continuous sleep while preserving sleep architecture by proportionally increasing REM and non-REM sleep stages.	1	100%	8.71	0.49	9
B. Daytime Functionality						
4	Improving daytime functionality should be considered a key therapeutic goal in the treatment of chronic insomnia, as it can have a direct positive impact on patient quality of life.	1	100%	8.86	0.38	9
In cases of chronic insomnia without associated comorbidities, improvement in nighttime sleep may be accompanied by improvements in the following daytime functionality impairments:						
5	– Fatigue	2	100%	9.00	0.00	9
6	– Sleepiness	2	100%	9.00	0.00	9
7	– Mood disturbances	2	100%	8.71	0.49	9
8	– Irritability	2	100%	8.71	0.49	9
9	– Attention and concentration deficits	2	100%	8.86	0.38	9
10	– Sensation of instability and/or clumsiness	2	100%	8.71	0.49	9
11	Improving daytime functionality should be considered one of the primary objectives along with improving the quality and quantity of nighttime sleep.	1	100%	8.43	0.53	8
C. Sleep Architecture						
12	Preservation of physiological sleep stages should be considered a therapeutic goal in the treatment of insomnia.	2	100%	8.57	0.53	9
13	Restoring a physiological sleep pattern (N1-N2-N3-REM) is important for improving sleep quality and is associated with better clinical outcomes (improved daytime functionality, reduced sleepiness, and better mood) compared to simply increasing total sleep duration.	1	100%	8.57	0.53	9
Improving sleep architecture may provide additional benefits in terms of:						
14	– Memory consolidation	2	100%	9.00	0.00	9
15	– Alertness and cognitive capacity	2	100%	9.00	0.00	9
16	– Mood	2	100%	8.86	0.38	9
17	– Immune system	2	100%	8.71	0.76	9
18	– Development and progression of comorbidities	2	100%	8.43	0.79	9
In chronic insomnia, maintaining sleep architecture during treatment should be considered:						
19	– Short-term	2	100%	8.57	0.79	9
20	– Long-term	2	100%	8.57	0.79	9
D. Abuse Potential and Addiction Risk of Dual Orexin Receptor Antagonists (DORAs)						
21	DORAs, which belong to a new pharmacotherapeutic group of non-sedative hypnotics (ATC N05CJ), have demonstrated efficacy in reducing wakefulness without causing sedation or nonspecific CNS effects.	1	100%	8.57	0.53	9
22	DORAs present a low potential for abuse/addiction compared to traditional hypnotics (benzodiazepines and Z-drugs).	2	100%	8.71	0.49	9
23	Since there is no evidence of addiction, discontinuation of DORA treatment has not been associated with withdrawal symptoms.	2	100%	8.71	0.49	9
24	In patients on long-term DORA treatment, periodic clinical monitoring specifically aimed at detecting possible signs of tolerance or non-therapeutic use is not considered necessary.	2	85.7%	7.86	2.27	9
Discontinuation of DORA treatment is associated with a low incidence of:						
25	– Withdrawal symptoms	2	100%	8.71	0.49	9
26	– Rebound insomnia	2	100%	8.71	0.49	9

CNS, central nervous system; DORAs, dual orexin receptor antagonists; GABA, gamma-aminobutyric acid; OX1R and OX2R, orexin receptor type 1 and type 2; REM, rapid eye movement; SD, standard deviation.

**Table 4:** Consensus statements on the clinical use of daridorexant.

#	Statement	Phase (consensus reached)	%	Mean	SD	Median
A. Comparative Clinical Benefit of Daridorexant						
In clinical practice, compared to other pharmacological treatments for insomnia, daridorexant shows:						
1	– Superior clinical benefit (objective and subjective sleep parameters, including daytime functioning)	2	100%	8.29	0.49	8
2	– A better safety and tolerability profile	2	100%	8.71	0.49	9
3	In clinical trials, daridorexant 50 mg increases the proportion of patients with insomnia who experience clinically meaningful improvements in daytime functioning each week, compared to placebo.	1	100%	8.57	0.53	9
4	Patients with insomnia treated with daridorexant 50 mg perceive an improvement in daytime functioning that increases week by week.	1	100%	8.29	0.76	8
5	Daridorexant restores and preserves sleep architecture in a more physiological manner than traditional hypnotics (benzodiazepines, Z-drugs, or sedative antidepressants).	1	100%	8.71	0.49	9
6	Daridorexant is associated with fewer cognitive adverse effects than traditional hypnotics (benzodiazepines, Z-drugs, or sedative antidepressants).	1	100%	8.71	0.49	9
7	The likelihood of patients responding to daridorexant treatment is greater than the likelihood of experiencing adverse effects such as somnolence, headache, or fatigue, indicating a favourable benefit-risk profile.	1	100%	8.57	0.53	9
B. Optimal Duration of Daridorexant Treatment						
8	In the treatment of chronic insomnia in adults, daridorexant is considered the drug of choice by clinicians, both for short-term and long-term treatment (> 4 weeks).	2	100%	8.29	0.76	8
9	Daridorexant should be maintained for at least 3 months to achieve sustained clinical benefits.	1	100%	8.71	0.49	9
10	The optimal duration of daridorexant treatment should be tailored to each patient’s characteristics and clinical course.	1	100%	8.57	0.53	9
Real-world long-term outcomes (up to 12 months) with daridorexant support the available scientific evidence regarding:						
11	– Its efficacy in the treatment of chronic insomnia	2	100%	8.71	0.49	9
12	– Its safety and tolerability	2	100%	8.86	0.38	9
13	– Its potential to improve both nocturnal sleep and daytime quality of life	2	100%	8.71	0.49	9
C. Objective versus Subjective Variables and Adherence						
The benefits perceived by patients usually align with the following objective sleep improvements:						
14	– Increased total sleep time (TST).	1	100%	8.57	0.53	9
15	– Reduced wakefulness after sleep onset (WASO).	1	100%	8.57	0.53	9
16	Treatment decisions with daridorexant should be based on standardised and/or subjective sleep measures (sleep diary, Insomnia Severity Index [ISI]).	2	100%	8.71	0.49	9
17	The correlation between improved daytime functioning and sleep quantity and quality supports the benefits of daridorexant in relation to both nighttime and daytime symptoms in patients with chronic insomnia disorder.	1	100%	8.57	0.53	9
18	Aligning patient expectations with measurable outcomes improves adherence to daridorexant treatment.	1	100%	8.71	0.49	9
19	Patients’ perception of sleep improvement may favour greater treatment adherence.	2	100%	8.71	0.49	9
D. Responder Patient Profile						
20	Based on current real-world clinical experience, no specific patient profile has been identified as responding better to daridorexant.	2	100%	8.71	0.49	9
21	In addition to patient profile, other factors (family, lifestyle, etc.) should be considered when predicting the response to daridorexant.	1	100%	7.71	0.76	8
When initiating and monitoring daridorexant treatment for chronic insomnia, the following assessments are essential:						
22	– Accurate diagnosis	1	100%	8.86	0.38	9
23	– Presence of comorbidities	1	100%	8.71	0.49	9
24	– Concomitant medications	1	100%	9.00	0.00	9
E. Switching Between Medications						
25	When switching to daridorexant, no initiation or withdrawal titration is required, as the recommended dose is fixed: 50 mg.	2	100%	9.00	0.00	9
26	No washout period is necessary when switching from hypnotics (benzodiazepines or Z-drugs) to daridorexant.	2	100%	8.57	0.53	9
Factors to consider when initiating daridorexant (direct start or during medication switch):						
27	– Type, duration, and dose of previous treatment	1	100%	8.29	0.76	8
28	– Patient expectations (sleep improvement, daytime functioning, and drug safety)	1	100%	8.57	0.53	9
Recommendations for transitioning from benzodiazepines, Z-drugs, or other hypnotic-sedatives to daridorexant:						
29	Gradual reduction of benzodiazepines, Z-drugs, or other hypnotic-sedatives (e.g., 10–25% decrements every 2–3 weeks) to minimise withdrawal symptoms.	2	100%	8.86	0.38	9
30	Individualised tapering schedule (percentage, intervals, and total duration) based on the patient’s clinical response (insomnia control, adverse effects or withdrawal symptoms, and improvement in daytime functioning).	2	100%	8.86	0.38	9

SD, standard deviation.

**Figure 2:**
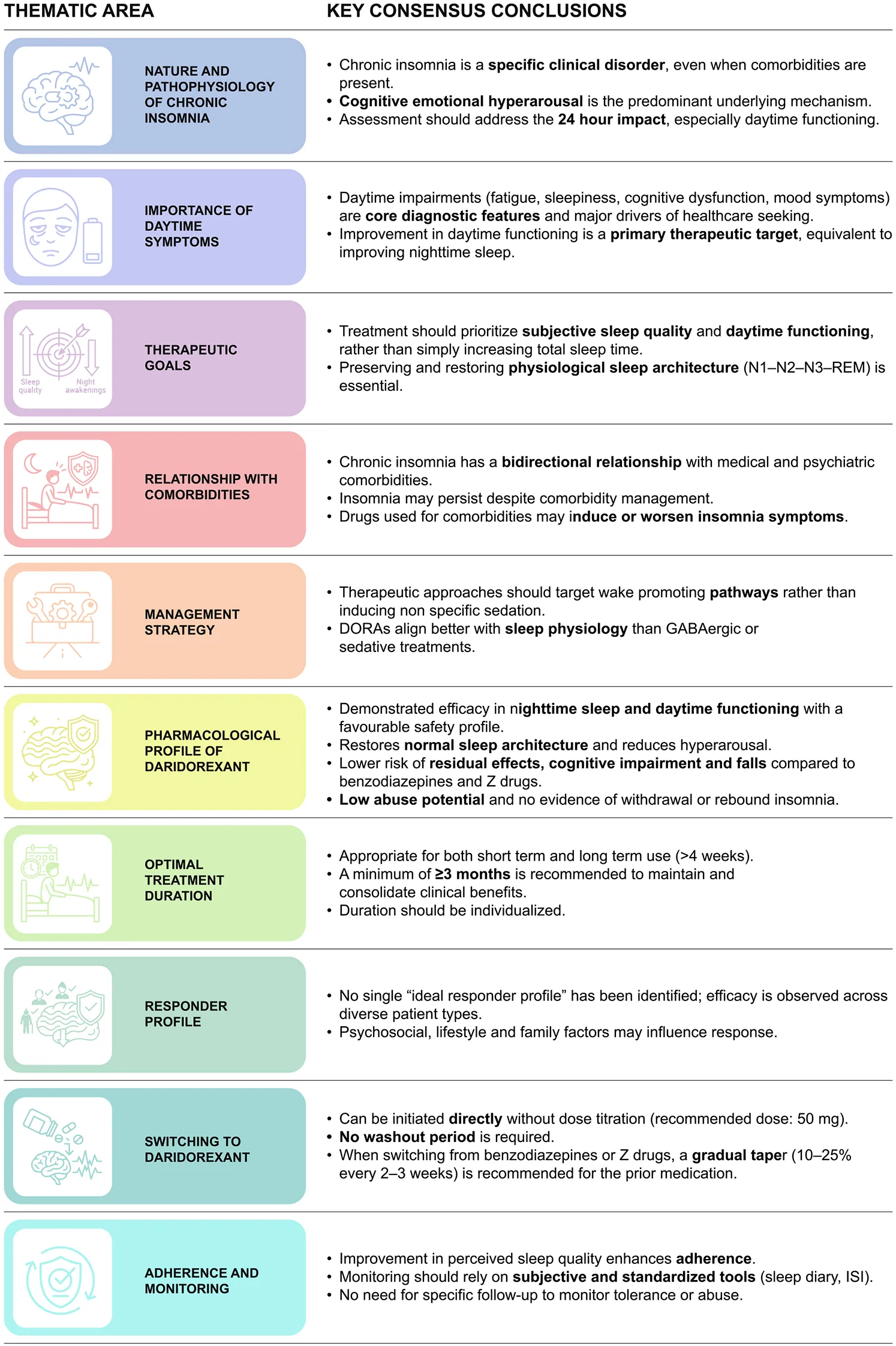
Key conclusions from the spanish expert consensus on chronic insomnia and daridorexant. DORAs, dual orexin receptor antagonists; GABA, gamma-aminobutyric acid; ISI, insomnia severity index; REM, rapid eye movement.

### 3.1 Nature, pathophysiology, and diagnostic approach of chronic insomnia

#### 3.1.1 Chronic insomnia as an independent disorder

The statements regarding the nature of chronic insomnia are listed in **Table 2** (Nos 1–4). The panel’s unanimous agreement that chronic insomnia is a specific disorder (**Table 2**) is consistent with current diagnostic classifications and contemporary clinical evidence. ICSD-3 (5), DSM-5 (36) and ICD-11 (4) all define chronic insomnia disorder using specific criteria relating to frequency, duration and daytime impairment, and allow for a diagnosis even when medical or psychiatric comorbidities are present. This supports its status as an independent condition. Furthermore, recent clinical documents show that chronic insomnia is common and functionally impairing and is associated with healthcare utilisation and adverse outcomes (1, 14). This underlines the importance of specific diagnostic pathways and targeted treatments, rather than viewing chronic insomnia solely as a symptom of other disorders, as this can lead to suboptimal management (27, 37).

There was also unanimous agreement that daytime impairments such as fatigue, excessive sleepiness, cognitive or functional impairments, and mood disturbances are central to the disorder and constitute a major reason for seeking medical attention (**Table 2**). Meta-analyses consistently link insomnia to cognitive and occupational deficits, emphasising the importance of daytime effects in the disorder (38). These results highlight the importance of systematically assessing daytime functioning and framing chronic insomnia as a 24-hour disorder that affects both night-time sleep and daytime performance. This perspective aligns with recent reviews emphasising the continuous night-day impact of insomnia on cognitive, emotional and functional domains (39). Recent evidence supports this perspective: daytime symptoms of insomnia, which were historically underrepresented in treatment and research (40), are increasingly recognised as core features (41–45). In patients with chronic insomnia treated with daridorexant, improvements in nocturnal sleep parameters, particularly reductions in night-time wakefulness, were correlated with better daytime functioning (42). Despite growing interest in daytime outcomes, standardised measurement remains limited. Most studies rely on self-report instruments, which vary in quality and are prone to bias, while objective assessments are rare and inconsistent (38). Emerging tools like wearable technology and digital phenotyping offer alternatives for capturing behavioural rhythms, mobility, and alertness, but raise ethical concerns (46).

#### 3.1.2 Hyperarousal mechanisms

Statements regarding hyperarousal as an underlying mechanism are listed in **Table 2** (Nos 5–7). Unanimous agreement that cognitive and emotional hyperarousal represents the predominant underlying mechanism of chronic insomnia reflects its broad acceptance as a central pathophysiological feature. This predominance is reinforced by objective neurophysiological markers indicating that chronic insomnia is characterised by persistent intrusions of wake-like activity during sleep. Large-scale polysomnographic analyses demonstrate increased transition probabilities from any sleep stage to wakefulness, elevated alpha and theta activity during wakefulness and N1 sleep, reduced delta power and diminished spindle density. These factors converge to create a state of sustained cortical activation that is incompatible with normal sleep regulation (47). These findings are in line with contemporary neurobiological models that describe insomnia as a disorder involving heightened activity in the arousal system. This hyperactivity affects stress-responsive and orexinergic pathways, as well as impairing GABAergic inhibition and leading to maladaptive neuroplasticity within corticolimbic circuits that are crucial for emotional regulation (48).

There was agreement that hyperarousal should be assessed as part of the diagnostic evaluation, but this was not universal (85.7%). This highlights the operational challenges involved, including the limited availability of validated tools and methodological heterogeneity. The European Insomnia Guideline explicitly states that polysomnography (PSG) should not be used routinely in chronic insomnia but rather reserved for cases in which another sleep disorder is suspected, or for treatment-resistant insomnia. This is due to its high resource demands and limited utility in modifying diagnostic impressions in typical insomnia presentations (1). Similarly, actigraphy is not recommended for routine insomnia evaluation as its sleep–wake classification algorithms cannot reliably distinguish between sleep and quiet wakefulness, an especially relevant limitation in insomnia, and therefore its role is restricted to specific differential diagnostic contexts (1). The difference between the estimated prevalence of insomnia and the capacity of sleep units is clear. Few multidisciplinary centres offer comprehensive evaluation and specific treatments, and referral programmes usually manage complex cases after years of insomnia. This means that simple tests or pharmaceutical treatment are prioritised over complex assessments or structured therapy (3, 49).

In addition, the panel unanimously agreed that hyperarousal may explain limited response to traditional hypnotics in some patients, highlighting the need for therapeutic strategies that directly target cognitive–emotional activation. This interpretation is consistent with current psychoneurobiological models of insomnia, which show that persistent cognitive, emotional and cortical hyperactivation is not adequately addressed by conventional GABAergic hypnotics, thereby contributing to suboptimal treatment responses (1).

#### 3.1.3 Sleep quality versus quantity as a therapeutic goal

All statements regarding therapeutic goals achieved unanimous consensus, with high mean scores indicating that nocturnal and daytime outcomes are both considered important treatment targets (**Table 2**; Nos 8–12). Improvement in sleep quantity (e.g. total sleep time) was rated as highly relevant (mean 8.6), but even higher scores were observed for perceived sleep quality (how restorative and refreshing sleep is) and daytime functioning. These high ratings for subjective quality and daytime functioning align with evidence showing that subjective sleep quality is weakly predicted by PSG. Studies demonstrate that patients often report non-restorative sleep despite normal objective parameters, highlighting a core discrepancy in insomnia assessment (50–54). The chronic use of sedative-hypnotics in older adults further illustrates this, as it extends sleep duration yet disrupts sleep architecture (e.g. reduced slow-wave sleep and theta power) and brain oscillations, yielding poorer daytime outcomes despite apparent gains in sleep quantity (55). Phase 3 clinical trials of novel DORAs demonstrate improvements in both patient-reported quality and functioning, as well as in sleep architecture, often with modest macrostructural changes. This further reinforces the importance of these factors (56). Ideally, treatments for insomnia should cover the 24-hour symptomatology to ensure effectiveness throughout the night and optimise daytime functioning. Overall, these findings advocate the systematic use of patient-reported outcome measures and composite indices that integrate subjective quality with physiological metrics, in order to evaluate insomnia treatments not only by their impact on objective parameters, but also by their ability to enhance perceived restorative sleep and daytime functioning.

#### 3.1.4 Comorbidities and bidirectional interactions

The panel unanimously agreed that chronic insomnia is a risk factor for medical and psychiatric comorbidities, which can exacerbate insomnia, creating a dynamic, bidirectional relationship. They also agreed that insomnia symptoms can persist despite adequate treatment of comorbid conditions, and that pharmacological agents used for such comorbidities may trigger or aggravate insomnia (**Table 2**; Nos 13–17).

Among sleep-related comorbidities, comorbid insomnia and sleep apnea (COMISA) represents a common and frequently underrecognised clinical condition. Emerging evidence suggests that COMISA is associated with greater symptom burden, poorer quality of life, and increased complexity of management compared with either disorder alone. Recognising COMISA may facilitate more integrated, personalised management strategies that address both disorders and improve the translation of current evidence into routine clinical practice (57, 58).

Longitudinal data demonstrate that insomnia independently increases the risk of developing conditions such as hypertension, coronary heart disease, depression and anxiety, and that these conditions can, in turn, precipitate or perpetuate insomnia (48, 59–63). For instance, a 12-month longitudinal cohort study conducted during the pandemic found that new-onset and pre-existing insomnia were predictive of persistent anxiety and depressive symptoms, even when psychiatric history was considered (64). Similarly, meta-analytic data confirm that insomnia more than doubles the risk of future depression (relative risk ≈2.3) (65). Meta-analytic evidence further demonstrates that insomnia significantly predicts suicidal ideation, suicide attempts and suicide mortality, even after controlling for psychiatric comorbidities. This highlights its role as a severe independent risk factor (66). Furthermore, insomnia often remains refractory even when underlying disorders are managed, and certain medications such as antidepressants, corticosteroids, and beta-blockers can exacerbate sleep disturbances (67).

These multiple lines of evidence support a model in which chronic insomnia and comorbidities impact each other over time, requiring management of both using appropriate, indication-approved treatments, and representing a substantial burden for patients and healthcare systems (14, 68). As previously discussed, recognising insomnia as a distinct therapeutic target rather than merely a symptom is in line with current guidelines and epidemiological findings and is crucial for improving long-term outcomes.

### 3.2 Management strategies in chronic insomnia

#### 3.2.1 Targeting wakefulness versus inducing sedation

Experts unanimously endorsed the physiological rationale that selective inhibition of the orexinergic pathway facilitates sleep onset and sleep maintenance by targeting wakefulness mechanisms rather than inducing sedation. This approach was rated as highly appropriate, emphasizing preservation of sleep architecture and differentiation from GABAergic or other nonspecific sedative pathways. The panel agreed that treatment of chronic insomnia should aim to modulate wake-promoting systems to ensure continuity and quality of sleep while maintaining normal rapid eye movement (REM) and non-REM cycling (**Table 3**; Nos 1–3).

Selective inhibition of the orexinergic pathway is consistent with current models of the pathophysiology of chronic insomnia disorder. In these models, orexin neuropeptides, also known as hypocretins, stabilise wakefulness and contribute to stress-related arousal dysregulation (69). Given the pivotal role of orexins in activating orexin receptors 1 and 2 (OX1R and OX2R) to promote and sustain wakefulness, antagonising these receptors is a rational and targeted strategy for improving sleep parameters. This mechanism facilitates sleep onset and maintenance by modulating wake-promoting systems rather than inducing diffuse GABAergic sedation. This offers a novel, differentiated treatment paradigm compared with traditional hypnotics that act on GABAergic, histaminergic or melatoninergic pathways (16, 17, 69, 70). Agents with affinity for both OX1-R and OX2-R are classified as DORAs (16), and have been shown to significantly improve sleep latency, sleep maintenance, total sleep time and sleep efficiency (56, 71–73). Interestingly, DORAs primarily increase REM sleep, which contrasts with other hypnotics, such as Z-drugs, BZDs, barbiturates and sedative antidepressants, that typically increase non-REM sleep while delaying or reducing REM onset (74, 75). Pooled phase 3 analyses with daridorexant further support this differentiated profile, showing reduced wake-to-wake transitions, increased transitions from wake to N1/N2/REM, attenuation of beta power during wake/N1 without changes in N2/N3/REM spectral bands or spindle metrics, and selective reductions in long wake bouts associated with improved daytime functioning (56, 72).

#### 3.2.2 Daytime functioning

Complete consensus was reached across all items (**Table 3**; Nos 4–11), highlighting daytime functionality as a central therapeutic outcome. Improvements in fatigue, sleepiness, irritability, attention, mood disturbances and the sensation of instability were consistently rated as having the highest mean scores (8.7–9.0). Importantly, daytime symptoms are not only common but are also considered essential for diagnosis according to DSM-5 criteria (36), yet they remain underrepresented in both research and treatment strategies. This imbalance is reflected in the literature, where most randomised controlled trials of insomnia interventions prioritise sleep onset latency, wake after sleep onset and sleep efficiency rather than functional outcomes during waking hours (45). As discussed earlier, there is still a lack of standardised, reliable, and validated tools for assessing daytime symptoms in populations with insomnia. However, recent evidence is helping to bridge this gap. *Post-hoc* analyses from daridorexant phase-3 trials demonstrate weekly improvements (earlier and sustained vs placebo) in scores on the validated instrument, the Daytime Symptoms and Impacts Questionnaire (IDSIQ), which was developed in accordance with FDA guidance (76). These scores correlate with self-reported sleep time and morning sleepiness (42). Dose-response analyses further indicate efficacy at 50 mg with less somnolence/fatigue, including daytime functioning gains (77). Beyond pharmacotherapy, a randomised trial comparing behavioural therapy with zolpidem in people with depression showed equivalent short-term improvements in depressive symptoms, fatigue, functional impairment and mental health, with added benefits from second-stage psychological therapy, underscoring the need to measure and target daytime functioning in trial designs (78). The field is advancing towards better measurement. The IDSIQ is proving sensitive to clinically meaningful change across the domains of alertness, cognition, mood, and sleepiness, while wearables and digital phenotyping enable the objective tracking of behavioural rhythms, mobility, and alertness. However, there are limitations in concordance with subjective impairment, and there are concerns around privacy and algorithmic bias (45, 46, 76). These developments reinforce the panel’s position that enhancing daytime functioning should be considered an equivalent therapeutic priority to nighttime sleep improvement. Future studies should prioritise multimodal assessment strategies that integrate validated self-report instruments (e.g. IDSIQ), performance-based behavioural tasks and passive sensing technologies, in order to yield comprehensive and realistic evaluations of the daytime consequences of insomnia across various functional domains.

#### 3.2.3 Preservation of physiological sleep architecture

Preservation of physiological sleep architecture (N1–N2–N3–REM stages) emerged as an unanimously endorsed core treatment goal in expert consensus, underscoring its crucial role in ameliorating subjective sleep quality alongside enhancements in cognitive performance, mood stabilisation, immune function, and slowing the comorbidity progression (**Table 3**; Nos 12–20). This prioritisation is consistent with evidence showing that BZD use, particularly in chronic or high-dose contexts, disrupts both macro- and microstructure. This occurs by increasing N2 while suppressing slow-wave sleep (N3) and REM, thereby producing a misleading sense of symptomatic improvement while degrading restorative processes (79, 80). Conversely, DORAs, specifically daridorexant, have been shown to preserve physiological stage distribution by increasing both non-REM and REM sleep while reducing wake-after-sleep-onset and wake-to-wake transitions. They have also been shown to attenuate cortical hyperarousal without altering N2, N3 or REM spectral integrity (56). These pharmacodynamic advantages are further reinforced by meta-analytic data in obstructive sleep apnea populations, where DORAs improve total sleep time and efficiency without exacerbating the apnea–hypopnoea index or reducing oxygen saturation. This indicates respiratory safety in patients with comorbid insomnia and obstructive sleep apnea (81). Finally, intensive longitudinal monitoring studies show that objective continuity, duration and stage composition, particularly depth in N3 and stability of REM, robustly predict nightly subjective sleep perception (82). This emphasises that long-term clinical improvement depends on restoring sleep architecture rather than inducing non-specific hypnotic sedation.

#### 3.2.4 Abuse potential and addiction risk of orexin receptor antagonists (DORAs)

All statements regarding the safety profile and low abuse potential of DORAs reached high consensus among experts (**Table 3**; Nos 21–26). This perception aligns with clinical evidence across the class, where DORAs selectively block OX1R and OX2R receptors in wake-promoting centres, reducing orexinergic activity without depressing the nervous system, unlike GABAergic hypnotics. Phase 3 trials of daridorexant demonstrated efficacy in reducing wake after sleep onset and sleep onset latency, with good tolerability and no rebound insomnia or withdrawal upon discontinuation (21). Long-term extension data confirm its tolerability up to 12 months without tolerance development (20), while lemborexant studies show sustained efficacy over 12 months without rebound during follow-up or withdrawal signals (83). Suvorexant similarly maintained efficacy in a 1-year phase 3 trial with safe abrupt discontinuation (84). Human abuse potential studies in recreational sedative users report daridorexant with lower “drug-liking” scores than zolpidem and suvorexant at clinically relevant doses, maintaining a favourable profile even supratherapeutically (85). Preclinical data reinforce this, as neither suvorexant, daridorexant nor lemborexant induced self-administration or reinforcement behaviours in animal models (86–88). Additionally, recent FAERS-based analyses identified primarily mechanism-related events (e.g., nightmares and hangover-like sensations) as infrequent safety signals across several years of post-marketing experience (89, 90), which supports the expert consensus regarding the limited need for long-term monitoring on this regard. Nevertheless, ongoing clinical follow-up remains important to evaluate treatment response, tolerability, and individual patient circumstances in routine practice.

### 3.3 Clinical evidence and therapeutic profile of daridorexant

#### 3.3.1 Comparative clinical benefits

There was full consensus that daridorexant provides superior clinical benefits compared with other pharmacological treatments for insomnia, encompassing both objective and subjective sleep parameters as well as improvements in daytime functioning, according to the judgement of the expert panel, in the absence of direct comparative evidence. Experts agreed that daridorexant has a more favourable safety and tolerability profile, and that there is supporting evidence showing that the 50 mg dose progressively enhances daytime functioning over the weeks of treatment. Daridorexant was also recognised by the expert panel as having characteristics consistent with the preservation of physiological sleep architecture and a favourable cognitive tolerability profile compared with BZDs, Z-drugs, or sedative antidepressants. Overall, the panel concluded that the likelihood of a clinical response to daridorexant outweighs the probability of experiencing mild adverse events such as somnolence, headache or fatigue, confirming its distinctly favourable benefit–risk profile (**Table 4**; (Nos 1–7).

These results align with a substantial body of evidence from phase 3 randomised controlled trials and *post hoc* analyses demonstrating the efficacy, safety and long-term tolerability of daridorexant in patients with chronic insomnia disorder (19, 20, 91, 92). However, it should be noted that most available evidence is derived from placebo-controlled studies, whereas direct head-to-head comparisons with benzodiazepines, Z-drugs, or sedating antidepressants remain limited. Pooled analyses have confirmed improvements in sleep parameters and daytime functioning (56), while meta-analyses have reinforced the advantages of daridorexant compared with traditional hypnotics (93, 94). They have also shown that its efficacy is comparable to other DORAs, such as lemborexant and suvorexant, although indirect analyses suggest numerically higher rates of residual daytime somnolence with lemborexant and suvorexant, and moderate pharmacokinetic accumulation with repeated lemborexant dosing (95, 96). Recent real-world evidence further support these findings, with studies showing clinically meaningful improvements in sleep quality, daytime alertness, and overall treatment satisfaction in patients treated with daridorexant in routine clinical practice (18, 23, 25, 26).

Studies assessing sleep architecture have shown that daridorexant improves sleep continuity by reducing hyperarousal markers and increasing transitions into non-REM and REM sleep, while preserving the normal architecture of REM and slow-wave sleep (56). Furthermore, comparative benefit–risk evaluations demonstrate an optimal balance between the number needed to treat and the number needed to cause harm (97). These findings are consistent with those of other Italian expert-based research which has also emphasised the clinical value, safety and positive impact of daridorexant on daytime functioning in real-world practice (33). Taken together, these data reaffirm daridorexant’s position as an effective, well-tolerated therapeutic option that addresses key unmet needs in chronic insomnia management.

#### 3.3.2 Optimal duration of daridorexant treatment

Panel consensus was unanimous across all statements regarding the optimal duration of daridorexant therapy for adults with chronic insomnia (**Table 4**; Nos 2–13). Experts agreed that daridorexant is considered an appropriate pharmacological treatment option for both short-term and long-term management (>4 weeks) of chronic insomnia in adults, with clinicians recommending at least 3 months of therapy to consolidate sustained clinical benefits. Evidence from randomized phase-3 programs and extensions substantiates this position: benefits on sleep onset, maintenance, and patient-reported daytime functioning are observed within the first month and maintained through month 3, particularly with 50 mg (21, 92). Long-term extension data show favourable tolerability and sustained effectiveness over 6–12 months (including older adults), without signals of physiological tolerance, rebound insomnia, or deterioration of sleep architecture (20, 24, 98, 99). Nevertheless, the panel agreed that the duration of treatment should be tailored to the individual patient based on their characteristics and the course of their condition. In addition, real-world long-term data (up to 12 months) were considered consistent with the existing scientific evidence regarding daridorexant’s efficacy, safety, and effects on both nocturnal sleep and daytime quality of life.

In addition to clinical trial and extension data, preliminary real-world data from three Spanish centres (N = 81) suggest that daridorexant is commonly prescribed for a mean duration of 5–6 months (100). This timeframe appears clinically reasonable in routine practice, particularly for consolidating the initial response and facilitating the transition from previous hypnotic therapies. These recently reported preliminary data are consistent with existing evidence regarding efficacy and tolerability and support an individualised approach to treatment duration.

#### 3.3.3 Objective versus subjective variables and adherence

Statements regarding objective-subjective variables and adherence to daridorexant treatment are listed in **Table 4** (Nos 14–19).

The experts agreed that patient-perceived benefits generally align with objective improvements in sleep parameters, including increased total sleep time (TST) and reduced wakefulness after sleep onset (WASO). This is consistent with responder and correlation analyses in patients treated with daridorexant, where changes in subjective sleep quality and daytime functioning closely paralleled objective improvements in sleep continuity and duration (42, 77). Evidence from patient-preference and benefit–risk studies further indicates that individuals with insomnia prioritise outcomes such as better sleep onset, fewer nocturnal awakenings, and improved next-day functioning, and that these perceived benefits strongly influence willingness to initiate and maintain pharmacological treatment, as well as reducing risk of withdrawal (101). In this context, treatment decisions should integrate standardized objective and subjective assessments, including sleep diaries and ISI, alongside actigraphy or PSG, despite their difficulties of access, to capture both measurable changes and patient-reported improvements (54, 102), reinforcing what was discussed earlier in Section 3.2.2 (Daytime functioning). Research on adherence shows that aligning therapy with the outcomes patients value most (e.g., daytime functioning) and communicating preference-concordant benefit–risk profiles are associated with sustained engagement with daridorexant; patient-centric quantitative benefit–risk assessments predict a higher net benefit versus placebo at both 25 mg and 50 mg, supporting ongoing use when perceived gains are evident (101, 103). Complementarily, in CBT-I, managing expectations, providing clear feedback on progress, and linking subjective gains to concrete sleep metrics are key determinants of long-term adherence (104, 105). Accordingly, the panel emphasized that aligning patient expectations with realistic, quantifiable outcomes and regularly communicating observed improvements enhances adherence; patients perceived improvement in sleep further promotes sustained engagement with daridorexant treatment.

#### 3.3.4 Responder patient profile

The experts concluded that daridorexant is suitable option for a broad range of clinical presentations, rather than for a specific profile of patients with chronic insomnia only and its effectiveness has been demonstrated across diverse clinical characteristics and therapeutic contexts. However, they also agreed that factors such as family, lifestyle and psychosocial context may influence the way in which patients respond to treatment. They also deemed accurate diagnosis, assessment of comorbidities, and review of concomitant medications essential during the initiation and monitoring of treatment (**Table 4**; Nos 20–24). This aligns with the available evidence, which shows consistent efficacy and good tolerability across heterogeneous clinical scenarios. This view is consistent with real-world data showing clinically meaningful improvements in sleep and daytime functioning in diverse patient groups, including individuals with long-standing insomnia, comorbid medical or psychiatric conditions, and prior experience of other hypnotics (18, 23). In a multicentre Japanese real-world study, daridorexant 50 mg improved total and item scores on the Athens Insomnia Scale (AIS) regardless of baseline insomnia severity and prior hypnotic exposure. Nearly half of the participants achieved an AIS total score of ≤5 by week 4 (indicative of adequate sleep). Notably, improvements were also observed in participants with psychiatric comorbidities (25). Additionally, a naturalistic study with 3-month follow-up reported sustained improvements in insomnia symptoms among patients with comorbid mental disturbances (e.g., depressive, bipolar, anxiety, and substance use disorders) treated with daridorexant 50 mg. There were also significant improvements in comorbid depressive, anxiety, manic, and suicidal risk symptoms. This reinforces daridorexant’s role as a well-tolerated option in complex cases often underrepresented in clinical trials (22). Similarly, an Italian consensus concluded that no unique patient profile benefits most from treatment. Nevertheless, experts highlighted practical scenarios in which daridorexant could be useful, such as for patients who cannot tolerate BZDs or Z-drugs, patients with mild-to-moderate coexisting anxiety symptoms and patients who need to minimise next-day residual effects (33).

#### 3.3.5 Switching between medications

The expert panel reached full consensus on recommendations for switching to daridorexant in patients with chronic insomnia (**Table 4**; Nos 25–30), emphasising the importance of considering patient-specific factors such as the type, duration and dose of prior treatment, as well as expectations regarding sleep improvement, daytime functioning and safety. Specifically, when transitioning to daridorexant, there is no requirement for titration, as the recommended dose by the European Medicines Agency is 50 mg, (although a 25 mg dose may be considered according to clinical judgement and individual patient characteristics) (106) or a washout period at the point of discontinuation as the drug seems not to be associated with withdrawal and rebound symptoms, as previously discussed (21). However, withdrawal symptoms depend on the discontinued drug. For patients transitioning from BZDs, Z-drugs, or other sedative-hypnotics, a gradual tapering strategy of 10–25% dose reduction every 2–3 weeks should be applied. This schedule (percentage reduction, intervals, and total duration) should be tailored according to the patient’s clinical response, including control of insomnia, adverse effects, withdrawal symptoms, and improvement in daytime functioning. These recommendations are consistent with contemporary European clinical practice guidelines for switching or deprescribing hypnotic medications for chronic insomnia, which state that daridorexant do not necessitate special discontinuing or switching protocols (34). These guidelines also strongly recommend cross-tapering or gradual tapering programmes from hypnotics rather than abrupt switching, as this approach minimises the risk of rebound insomnia and withdrawal symptoms while ensuring continuity of care.

In the Italian consensus, experts emphasised the importance of sleep psychoeducation in order to manage expectations effectively, explaining that improvements in sleep and daytime functioning occur gradually. They also recommended the systematic use of the ISI and sleep diaries to monitor treatment progress and optimise outcomes when switching to daridorexant (33).

## 4 Discussion and conclusions

This multidisciplinary RAND/UCLA consensus shows strong alignment between clinical experience and current evidence in chronic insomnia management. Experts agreed that chronic insomnia is a 24-hour disorder, reinforcing the need to assess treatment success through subjective sleep quality, restorative sleep, and daytime functioning rather than nocturnal metrics alone.

The panel favoured therapeutic strategies that modulate wake-promoting pathways and address hyperarousal while restoring and preserving physiological sleep architecture. Within this framework, daridorexant was consistently considered an appropriate option for both short- and long-term treatment. A minimum duration of approximately three months and a likely timeframe of 6 months was viewed as generally necessary to consolidate benefits, although treatment should be individualised according to patient characteristics and response.

This work has limitations. As in all expert-based methods, conclusions reflect the perspectives of a relatively small panel. However, this limitation is mitigated by the panel’s multidisciplinary composition and broad representation of different autonomous communities and healthcare settings. This includes both public and private centres, helping to reflect the diversity of real-world sleep care approaches across Spain. The RAND/UCLA approach is designed for areas with limited or heterogeneous evidence; therefore, some recommendations rely primarily on expert judgement. Although the study received industry funding, all ratings were completed independently and anonymously by the expert panel. Therefore, the recommendations should be interpreted within the context of the available evidence, expert clinical judgement, and the Spanish healthcare setting.

In conclusion, this consensus supports a pragmatic, physiologically coherent, and patient-centred approach to chronic insomnia in routine practice. The recommendations reflect the integration of the available evidence and expert clinical judgement, with daridorexant being considered by the panel an appropriate treatment option when CBT-I is unavailable, insufficient, or not feasible. By promoting recognition of chronic insomnia as an independent disorder requiring specific assessment and management, this consensus may help improve the identification of complex clinical presentations in routine practice. Future research should focus on integrated treatment strategies and multidisciplinary care pathways for patients with overlapping sleep disorders, as well as on Spanish real-world data, particularly on daytime functioning and long-term treatment trajectories, to further guide clinical implementation.

We would like to express our gratitude to María Yuste Parra from Evidenze Health Spain S.L.U. for her support as a scientific advisor and medical writer during the elaboration of this article.

## Data Availability

The raw data supporting the conclusions of this article will be made available by the authors, without undue reservation.
